# Calibration and validation of the rabbit model of electrolytic‐mediated arterial thrombosis against the standard‐of‐care anticoagulant apixaban

**DOI:** 10.1002/prp2.963

**Published:** 2022-06-09

**Authors:** Pancras C. Wong, Earl Crain

**Affiliations:** ^1^ Cardiovascular & Fibrosis Drug Discovery Biology Bristol Myers Squibb Company Princeton New Jersey USA

**Keywords:** anticoagulant, blood coagulation, factor Xa inhibitor, thrombosis, translation

## Abstract

Apixaban is a factor Xa (FXa) inhibitor and standard‐of‐care anticoagulant with FXa Ki and plasma protein binding (free fraction) averages 0.08 nM and 0.13 in humans and 0.16 nM and 0.37 in rabbits, respectively. Apixaban at the approved dose of 5 mg BID achieved maximum and minimum plasma concentration of 373 nM (95% CI: 198 – 699 nM) and 224 nM (95% CI 89–501 nM), respectively, in patients with nonvalvular atrial fibrillation (AF). We calibrated the rabbit model of electrolytic‐mediated arterial thrombosis (ECAT) against apixaban and correlated the potencies derived from the rabbit ECAT to in vivo efficacious exposure levels in AF patients. Vehicle and apixaban at multiple doses were infused IV in ECAT rabbits and their effects on thrombus weight were measured. Apixaban exhibited dose‐related efficacy in preventing thrombosis in ECAT rabbits with EC_20_, EC_50_, EC_60_, EC_70_ and EC_80_ of 18, 101, 169, 296, and 585 nM, respectively. After correcting for the human‐to‐rabbit potency based on FXa Ki and plasma protein binding, we estimated a rabbit‐equally‐effective plasma concentration of 157 and 259 nM to the trough and peak plasma concentration in AF patients treated with 5 mg BID of apixaban. These rabbit‐equally‐effective plasma concentrations matched well with the rabbit ECAT EC_60_ and EC_70_. This study supports the potential of the rabbit ECAT to predict in vivo therapeutic drug exposure of FXa inhibitors. Achieving human‐equally‐effective plasma concentrations to the rabbit ECAT EC_60_ and EC_70_ may produce clinical efficacy in patient populations like AF.

## Graphical abstract

The rabbit model of electrolytic‐mediated arterial thrombosis (ECAT) was calibrated against the standard‐of‐care anticoagulant apixaban. Reverse translation from human apixaban data to rabbit suggested achieving human‐equally‐effective plasma concentrations to the rabbit ECAT EC_60_ and EC_70_ may produce clinical efficacy in patient populations like nonvalvular atrial fibrillation.

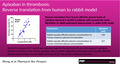

## 1 INTRODUCTION

Apixaban is a direct factor Xa (FXa) inhibitor and potent antithrombotic agent in pre‐clinical models.^1^, ^2^ Clinical studies suggest that apixaban provides consistent anticoagulation and a potentially optimal risk:benefit balance.^3^, ^4^ Indeed, the Apixaban for Reduction in Stroke and Other Thromboembolic Events in Atrial Fibrillation (ARISTOTLE) trial, a Phase 3 study, demonstrated that apixaban was superior to warfarin in reducing stroke or systemic embolism with less bleeding and lower mortality.^4^ Apixaban has now become a new standard‐of‐care anticoagulant for the prevention and treatment of thrombosis in several cardiovascular conditions and is marketed under the trade name of Eliquis.^5^

Animal models of thrombosis are valuable tools to study the biology of human thrombosis and testing new therapeutics.^6^ A major challenge in drug discovery is to establish viable translational animal models to predict the efficacy and doses of investigational drugs in support of studies in humans. We had used multiple rabbit models of thrombosis to evaluate the efficacies of small‐molecule director FXa inhibitors, which lead to the discovery of apixaban.^2^, ^7^ The rabbit model was selected because rabbit and human FXa have similar binding affinity to enzyme substrate and small‐molecule direct FXa inhibitors.^2^, ^8^ In contrast, rat and dog FXa are much less sensitive to small‐molecule direct FXa inhibitors.^2^, ^8^ A key model is the rabbit model of electrolytic‐mediated arterial thrombosis (ECAT). ^2^, ^7^ The rabbit ECAT model had been benchmarked with the standard‐of‐care antiplatelet agents, including aspirin and the P2Y12 antagonists, clopidogrel and prasugrel, as well as the standard anticoagulants, including fondaparinux and warfarin, and their antithrombotic efficacy profiles were similar to that reported in humans.^2^, ^9^

Byon et al., based on population‐pharmacokinetic models, estimated the apixaban steady‐state maximum and minimum plasma concentrations in patients with AF.^10^ This provides an opportunity to conduct a reverse translation from human data to rabbits, and examined the usefulness of the rabbit ECAT model to predict in vivo therapeutic drug exposure. To this end, we conducted a dose‐response study of apixaban in the rabbit ECAT model and correlated the potencies derived from the rabbit ECAT to in vivo clinically efficacious exposure levels in AF.

## 2 MATERIALS AND METHODS

### 2.1 Reagents

The following drugs and chemicals were used in this study: activated partial thromboplastin time (aPTT) reagent (Dade^®^ Actin^®^ FS Activated Reagent), prothrombin time (PT) reagent (Dade^®^ thromboplastin C Plus) and thrombin time (TT) reagent (Test Thrombin Reagent) from Dade Behring (Marburg, Germany). Apixaban was synthesized at Bristol Myers Squibb Company.^1^

### 2.2 Animals

Experiments were conducted in accordance with the NIH Guide for the Care and Use of Laboratory Animals, and the regulations of the Animal Care and Use Committee of the Bristol Myers Squibb Company.

### 2.3 Thrombosis studies

The rabbit ECAT model was used in this study.^7^ Briefly, male New Zealand White rabbits were anesthetized with ketamine (50 mg/kg + 50 mg/kg/h intramuscularly) and xylazine (10 mg/kg + 10 mg/kg/h intramuscularly). Thrombosis was induced by electrical stimulation of the left control carotid artery for 3 min at 4 mA using an external stainless‐steel bipolar electrode. Carotid blood flow (CBF) was measured with an electromagnetic flow probe continuously over a 90‐min period to monitor thrombosis‐induced occlusion. In addition, thrombus from the injured artery was removed, blotted twice on a weighing paper to remove residual fluid, and weighed.

After determination of the thrombus weight in the left control carotid artery, apixaban or its vehicle was given by an intravenous (IV) bolus injection supplemented with a continuous IV infusion 30 min before the electrical stimulation until the end of the experiment. The purpose of the bolus and infusion dosing protocol is designed to achieve a stable plasma level with minimum experimental variability. Thrombosis was then electrically induced in the right carotid artery, using the same method as mentioned above. Concentrations of apixaban in plasma samples were determined by the BMS Bioanalytical Research and were measured by a specific and sensitive liquid chromatographic mass spectrometry method (LC/MS/MS). ^1^, ^2^

The study consisted of vehicle (10% N,N‐dimethylacetamide:90% of 5% dextrose) and apixaban (mg/kg + mg/kg/h) at 0.018 + 0.026, 0.06 + 0.09, 0.18 + 0.26, and 0.6 + 0.87 (*n* = 6 per group).

### 2.4 Ex vivo coagulation assays

Arterial blood samples for the determination of ex vivo activated partial thromboplastin time (aPTT), PT and TT were collected in tubes containing one‐tenth the volume of 0.129 M sodium citrate before the start of the infusion of vehicle or test compound and at the end of the study. Clotting times were measured with an automated coagulation analyzer (Sysmex^®^, Dade Behring Inc., Deerfield, IL, USA) as previously described.^2^ The aPTT, PT, and TT reagents were reconstituted and assays were performed according to the manufacturer's instructions.

### 2.5 Statistical analysis

Statistical analyses used were ANOVA and Dunnett's test for multiple comparison using the GraphPad Prism version 8.4 for Windows. EC_20_ to EC_80_ doses were determined using the four‐parameter logistic equation and the logistic fit was analyzed by the GraphPad Prism version 8.4 for Windows (GraphPad Software, San Diego, CA, USA). A value of *p* < .05 was considered statistically significant. All data are means ± standard error of the mean (SEM).

## 3 RESULTS

**FIGURE 1:**
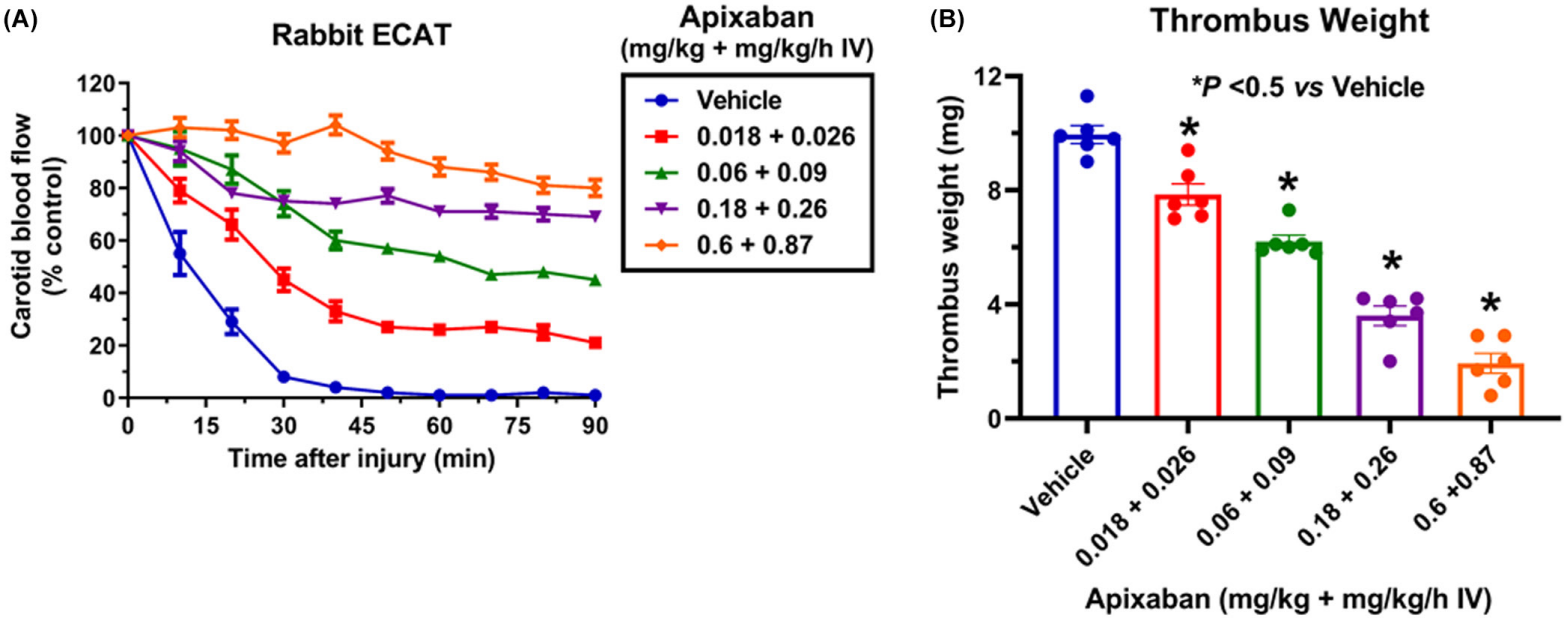
Antithrombotic effects in the rabbit model of electrically mediated carotid arterial thrombosis. (A) Effects of vehicle and apixaban at 0.018 + 0.026, 0.06 + 0.09, 0.18 + 0.26, and 0.6 + 0.87 (mg/kg + mg/kg/hr IV) (*n* = 6 per group) on carotid blood flow after thrombus induction in the injured carotid artery. Carotid blood flow was expressed as % of control carotid blood flow. (B) Effects of vehicle and apixaban at 0.018 + 0.026, 0.06 + 0.09, 0.18 + 0.26, and 0.6 + 0.87 (mg/kg + mg/kg/hr IV) (*n* = 6 per group) on thrombus weight. *p < .05, compared with vehicle. Data are means ± SEM

Basal CBF in the vehicle‐treated group (*n* = 6) averaged 16 ± 3 ml/min. After electric current stimulation, thrombosis formation occurred rapidly, resulting in reduction of CBF to 0 within 40–45 min in vehicle‐treated animals (Figure 1A). By blocking the formation of thrombus, apixaban was effective in preserving vascular patency in a dose‐dependent manner during the induction of occlusive thrombosis. Thrombus weight averaged 10.0 ± 0.3 mg following thrombus induction in vehicle‐treated animals. Apixaban at 0.018 + 0.026, 0.06 + 0.09, 0.18 + 0.26 and 0.6 + 0.87 mg/kg + mg/kg/h IV reduced thrombus weight significantly by 18 ± 3, 34 ± 1, 63 ± 3 and 78 ± 5%, respectively (*n* = 6 per group) (Figure 1B).

Effects of apixaban on ex vivo aPTT, TT, and PT were measured. aPTT, PT, and TT in the vehicle‐treated animals averaged 19.0 ± 1.4 s, 10.2 ± 0.3 s, and 22.5 ± 1.6 s, respectively. Effects (measured as fold‐increase) of vehicle and apixaban at 0.018 + 0.026, 0.06 + 0.09, 0.18 + 0.26, and 0.6 + 0.87 mg/kg + mg/kg/h IV on aPTT were 1.12 ± 0.07, 1.23 ± 0.02, 1.40 ± 0.05^*^ and 1.63 ± 0.10^*^, on PT were 1.08 ± 0.02, 1.12 ± 0.02, 1.37 ± 0.17^*^, 1.50 ± 0.04^*^, and on TT were 1.04 ± 0.02, 1.02 ± 0.03, 0.98 ± 0.03, 1.07 ± 0.04, respectively (*p < .05 vs. vehicle). As expected, apixaban did not prolong TT at all doses since it did not inhibit thrombin activity.^2^

**FIGURE 2:**
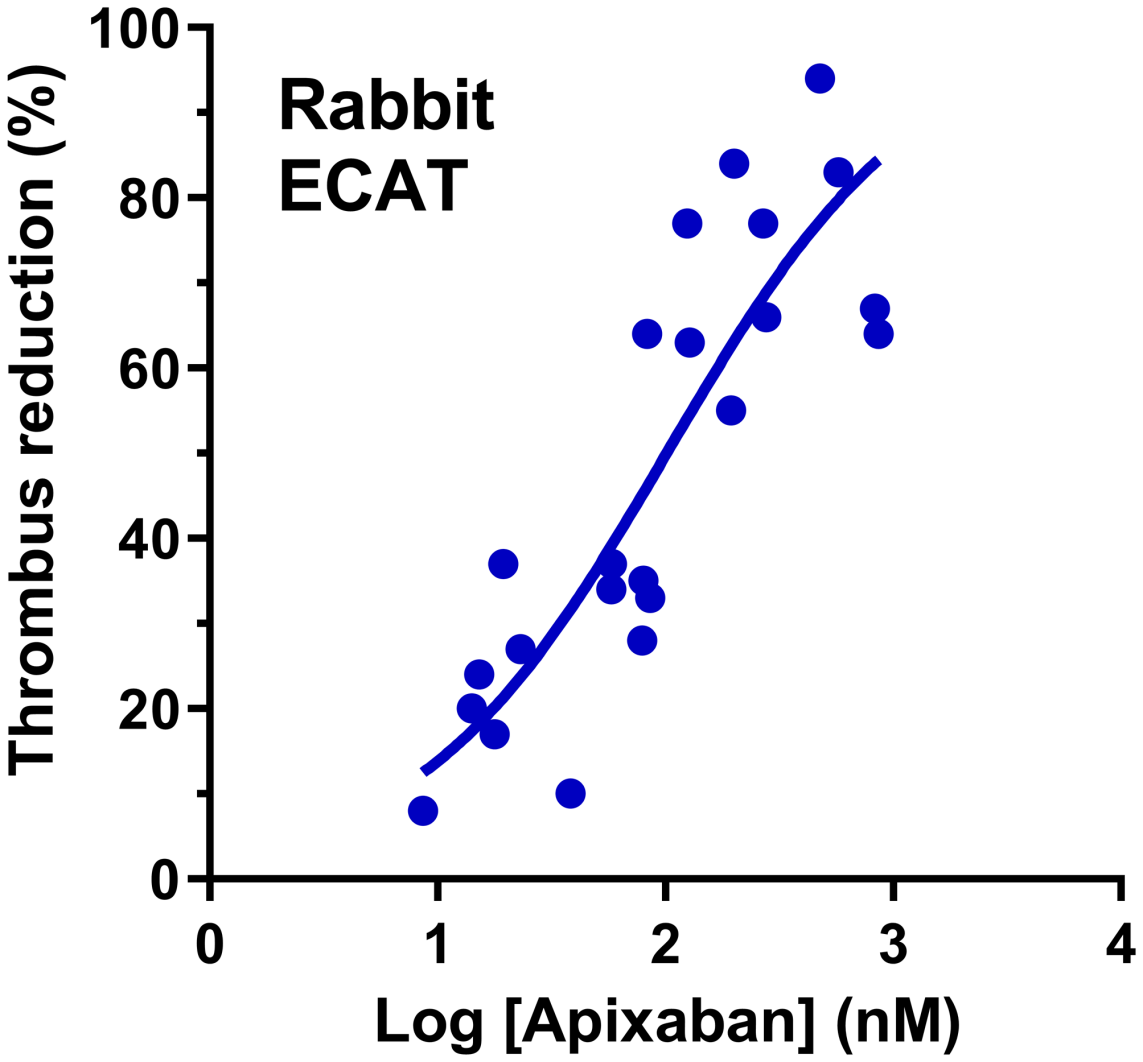
Relationship between total plasma concentration of apixaban and antithrombotic effects (expressed as a percentage reduction of the treated relative to the control thrombus weight) in rabbit electrolytic‐mediated arterial thrombosis model

Figure 2 shows the concentration‐response curve for apixaban in the ECAT model. Apixaban caused a concentration‐dependent antithrombotic effect with EC_20_ of 18 nM (95% CI = 13–25), EC_50_ of 101 nM (95% CI =71–146), EC_60_ of 169 nM (95% CI = 117 to 272), EC_70_ of 296 nM (95% CI = 186 to 580), EC_80_ 585 nM (95% CI =289–1187), and a hill slope of 0.8 (95% CI = 0.5–1.1) (*n* = 24). The EC_50_ value for apixaban reported in this study is similar to the EC_50_ value of 106 nM for apixaban reported previously.^2^

## 4 DISCUSSION

Based on simulations of population‐pharmacokinetic models, Byon et al. estimated the apixaban steady‐state maximum plasma concentration (C_max_) of 171 ng/ml (95% CI = 91–331) and minimum plasma concentration (C_min_) of 103 ng/ml (95% CI = 41–230) in patients with AF treated with 5 mg BID of apixaban to prevent stroke and systemic embolism.^10^ When expressed in molarity, the plasma concentration was 373 nM (95% CI: 198–699 nM) at C_max_ and 224 nM (95% CI: 89–501 nM) at C_min_ (Table 1). These clinical efficacy‐exposure data of apixaban provided us an opportunity to examine the usefulness of the rabbit ECAT model to project in vivo therapeutic drug exposure.

**TABLE 1:** Reverse translation from human effective plasma levels of apixaban dosed at 5 mg BID in patients with nonvalvular atrial fibrillation to rabbit‐equivalent plasma levels in the rabbit ECAT model

Species	Parameters	Apixaban
Human	FXa Ki (nM)	0.08^a^
Human	Plasma protein binding (free fraction)	0.13^a^
Human	Total maximum plasma concentration (nM)	373 nM^b^ (95% CI: 198–699 nM)
Human	Total minimum plasma concentration (nM)	224^b^ (95% CI: 89–501 nM)
Human	Free maximum plasma concentration (nM) after adjusting for plasma protein binding (free fraction)	48 (95% CI 26–91 nM)
Human	Free minimum plasma concentration (nM) after adjusting for plasma protein binding (free fraction)	29 (95% CI 12–65 nM)
Rabbit	FXa Ki (nM)	0.16^c^
Rabbit	Free rabbit‐equally‐effective plasma concentration (nM) to human maximum plasma concentration after adjusting for differences in Ki	96 (96% CI: 52–182)
Rabbit	Free rabbit‐equally‐effective plasma concentration (nM) to human minimum plasma concentration after adjusting for differences in Ki	58 (96% CI: 24–130)
Rabbit	Plasma protein binding (free fraction)	0.37^d^
Rabbit	Total rabbit‐equally‐effective plasma concentration (nM) to human maximum plasma concentration after adjusting for plasma protein binding (free fraction)	259 (95% CI: 141–492)
Rabbit	Total rabbit‐equally‐effective plasma concentration (nM) to human minimum plasma concentration after adjusting for plasma protein binding (free fraction)	157 (95% CI: 65–351)

^a^ Data from references [1, 10].

^b^ Data from reference [10].

^c^ Data from reference [2].

^d^ Data from reference [11].

Conversion of human target effect level to the rabbit equivalent target effect level requires the consideration of species differences in drug target binding and plasma protein binding. Apixaban exhibited species differences in FXa Ki and plasma protein binding.^1^, ^2^, ^10^ Its mean values of FXa Ki and plasma protein binding (free fraction) averaged 0.08 nM and 0.13 in human^1^, ^10^ and 0.16 nM and 0.37 in rabbits,^2^, ^11^ respectively.

As shown in Table 1, the total steady‐state maximum plasma concentration of apixaban at 5 mg BID was 373 nM in AF patients. The free maximum plasma concentration of apixaban, obtained via multiplying by the human plasma free fraction of 0.13, was about 48 nM. After adjusting for differences in FXa Ki between human (0.08 nM) and rabbit (0.16 nM), the free rabbit‐equally‐effective plasma concentration to human maximum plasma concentration was about 96 nM. Dividing the free rabbit‐equally‐effective plasma concentration by the rabbit plasma protein binding free fraction (0.37) yielded a rabbit total plasma concentration of 259 nM as an equally effective concentration to the human maximum plasma concentration (Table 1), which matched well with the rabbit ECAT EC_70_. A similar reverse translation from the total steady‐state minimum plasma concentration of apixaban of 224 nM in AF patients yielded a rabbit total concentration of 157 nM as an equally effective concentration to the human minimum plasma concentration (Table 1), which matched well with the rabbit ECAT EC_60_.

This study provided an example of reverse translation from clinical to preclinical for assessment of confidence in the rabbit ECAT model for drug discovery of new anticoagulants. It suggests that achieving human‐equally‐effective plasma concentrations to the rabbit ECAT EC_60_ and EC_70_ may produce clinical efficacy in patient populations like AF, and supports the potential of the rabbit ECAT to predict in vivo therapeutic drug exposures of new anticoagulants. To this end, we have on‐going preclinical and clinical studies with the Factor XIa inhibitor milvexian, a new class of anticoagulants, to further examine our approach of using the rabbit ECAT to project human efficacy and doses of milvexian.^12^

## DISCLOSURES

Authors are employee or former employee and stockholder of Bristol Myers Squibb Company. This work was supported by Bristol Myers Squibb and Pfizer.

## AUTHOR CONTRIBUTIONS

Pancras Wong contributed to the establishment of thrombosis model in rabbits, designed the study, supervised Earl Crain to perform the study, analyzed and interpreted the data, and wrote the manuscript. Earl Crain conducted animal studies and collected data from these studies. All authors approved the manuscript.

## ETHICS APPROVAL

Animal studies were conducted in accordance with the regulation and ethics of the Animal Care and Use Committee of the Bristol Myers Squibb Company.

## Data Availability

The data that support the findings of this study are available from the corresponding author upon reasonable request.
